# Retraction: Overexpression of Peanut Diacylglycerol Acyltransferase 2 in *Escherichia coli*

**DOI:** 10.1371/journal.pone.0252974

**Published:** 2021-06-04

**Authors:** The PLOS ONE Editors

Following the publication of this article [1], concerns were raised regarding results presented in Figs 1 and 7. Specifically,

- The background signal directly surrounding the bands presented in Fig 5B appears to be cut off at straight horizontal and vertical edges and appears to be inconsistent with the overall background of the blot.
- Fig 7 panels B, F, and H, demonstrate square and rectangular areas where the background colour and pattern does not appear to match the overall background signal of the rest of the panel. In addition, the background signal surrounding the cells in the Fig 7 A, C, D, E, and G panels appears to be cut off at straight horizontal and vertical edges and does not appear to match the overall background of these panels.

The first author indicated that the corresponding author is retired and that the raw data underlying the results presented in this study are no longer available.

In light of the concerns affecting multiple figure panels that question the integrity of these data, the *PLOS ONE* Editors retract this article.

The authors either did not respond to the editorial decision to retract this article or could not be reached.

## References

[1] Peng Z, Li L, Yang L, Zhang B, Chen G, Bi Y (2013) Overexpression of Peanut Diacylglycerol Acyltransferase 2 in *Escherichia coli*. PLoS ONE 8(4): e61363. 10.1371/journal.pone.0061363 23593473 PMC3623910

